# The Impact of Education Sources on Patient Compliance with the Recommended Oral Nutritional Supplement (ONS) Intake: A Qualitative Survey

**DOI:** 10.3390/nu17050889

**Published:** 2025-03-02

**Authors:** Natalija Uršulin-Trstenjak, Damir Poljak, Bojan Šarkanj, Melita Sajko, Ivana Dodlek Šarkanj

**Affiliations:** 1 Department of Food Technology, University North, Trg dr. Žarka Dolinara 1, HR-48000 Koprivnica, Croatia; bsarkanj@unin.hr (B.Š.); idsarkanj@unin.hr (I.D.Š.); 2 General Hospital Varaždin, Ivana Meštrovića 1, HR-42000 Varaždin, Croatia; dpoljak@unin.hr; 3 Department of Nursing, University North, 104. Brigade 3, HR-42000 Varaždin, Croatia; msajko@unin.hr

**Keywords:** oral nutritional supplements (ONSs), patients, education sources, compliance with ONS intake, statistically significant differences

## Abstract

Background: Nutritional support through oral nutritional supplements (ONSs) is important for patients. It leads to improved nutritional intake and better clinical and economic outcomes. Objectives: The problem that often accompanies the use of ONSs is their consumption below the optimal prescribed doses. One of the reasons for this is patient education on the importance of ONS intake. This study investigated hospitalized patients and focused on the impact that educational sources have on ONS intake. It also investigated patient compliance with the intake of the prescribed dose, and the time of initiation and discontinuation of ONS consumption. Methods: A qualitative survey using an anonymous questionnaire was conducted on 120 hospital patients across three locations in the Republic of Croatia: Varaždin General Hospital, the Special Hospital for Chronic Diseases in Novi Marof, and the hospital for lung diseases and tuberculosis in Klenovnik. Data were collected by independent nurses and analyzed using appropriate statistical tests, including Shapiro–Wilk, Levene, Mann–Whitney, and χ^2^ tests. Results: There is a statistically significant difference between compliance with the intake at home and the source of information on how to consume ONSs (*p* = 0.003). There is also a statistically significant difference between compliance with intake at home and the initiation of ONS consumption (*p* = 0.000) with a key time of six months (half a year) when most of them give up. Conclusions: Only 47.95% complied with the recommended dose of ONS intake at home who received the information from a nurse, if we look at the information source. There is a clear need to change patient education by developing a standardized form and tools accessible to patients. After six months, most patients discontinue ONS consumption. Further research is necessary to determine whether a medical specialist is the reason for discontinuation, or whether discontinuation happened due to insufficient education on the importance of ONS intake.

## 1. Introduction

Nutrition is recognized as a key element in disease prevention and the maintenance of good health. Illnesses can significantly affect nutritional status due to changes in nutritional needs, from intake and metabolism to excretion, with an increased occurrence of protein–energy malnutrition [1]. Alvarez-Altamirano et al. (2024) highlighted a high prevalence of malnutrition in a group of hospitalized patients in Mexico [2]. Hospital-acquired malnutrition constitutes a multifaceted and clinically significant issue in contemporary healthcare settings. This condition, characterized by inadequate nutritional status among hospitalized patients, has far-reaching implications for patient outcomes, healthcare resource utilization, and overall quality of care [3]. The importance of the personal nutritional status of elderly populations based on basic foodomics elements cannot be overstated in this context. Foodomics-based nutritional status assessment allows for the early identification of malnutrition risk in elderly populations, enabling healthcare providers to develop tailored dietary interventions that address specific deficiencies or imbalances. This personalized approach can lead to more effective nutritional support, potentially reducing the incidence and severity of hospital-acquired malnutrition in this vulnerable demographic [4]. Consequently, it has an impact on the quality of recovery and treatment strategy. Nutritional support, in the form of oral nutritional supplements (ONSs), is generally necessary for patients who cannot meet their nutritional needs through daily meals.

Scientific evidence shows that the use of ONSs can lead to improved nutritional intake, along with improved clinical, economic, and other outcomes [5]. Malnutrition in hospitalized patients is associated with increased morbidity, mortality, increased infection risks, and overall healthcare costs. The early identification and management of malnutrition is crucial, as it can lead to improved patient outcomes and a reduced length of hospital stay [6].

ONSs are available for clinical use as ready-to-use liquid or powder products rich in nutrients. They are classified into categories with the function of helping meet the nutritional needs of patients with various general medical conditions (e.g., malnutrition and frailty), while other ONSs are used for very specific conditions (e.g., emergency care) or diseases (e.g., diabetes and kidney disease) [7]. Not all ONS products are suitable for every patient. The composition of ONSs is nutritionally tailored to the patient’s needs [8].

ONSs in liquid form are of precisely defined compositions, small in volume (packaging), and ready for consumption with a reduced possibility of bacterial contamination. They come in a range of different flavors—chocolate, vanilla, coffee, strawberry, banana, forest fruit, etc. Therefore, it is advisable that a patient try them to see which flavor suits the person best because they need to be taken in fully prescribed doses (according to the doctor’s instructions).

During the first few days, consumption begins slowly, sip by sip, over a period of several hours, and later at intervals of 20–30 min, two hours before or after the main meals. The prepared supplement is usually more pleasant for consumption when it is slightly chilled, and it can also be diluted with water. Moreover, it can be an integral part of main meals, or used in various dishes heated to 40 °C. Once opened, the supplement can be stored in the refrigerator for 24 h or can be kept at room temperature for up to 2 h [8]. 

The problem that arises regarding ONS use is their intake below the optimal prescribed dose, both among hospitalized patients and in the continuation of intake at home [9]. 

One of the reasons for this is patient education on the importance of ONS intake [10]. 

There are four important components in patient education: assessment, planning, implementation, and evaluation. In order to achieve excellence, the nurse must implement each component in patient education [11]. It should be implemented individually based on patients’ characteristics and conditions, and it should be practical, objective, and based on patients’ mental abilities [12,13]. 

Nurses must be fully prepared to devote the necessary time and energy to patient education, and they should also believe in the consequent success. However, there are a number of problems that nurses encounter regarding patient education. One of them is the lack of adequate space for education due to high fluctuations in their departments, as well as the help of technology (video, TV, video software) for showing various educational films about nutrition and medication while patients are in their hospital beds. The shortage of nurses is also an unavoidable fact [14]. It is recommended that nurses undergo frequent professional training to improve the knowledge necessary for effective patient education [15,16]. At the same time, the conclusions of some studies speak in favor of monitoring patient education and providing constructive feedback, which represents a significant benefit for both nurses and patients [17].

Additional methods could be used to enhance the patient uptake of the ONSs. The teach-back method, which involves asking patients to explain instructions in their own words, has been shown to improve knowledge retention, self-care abilities, and health outcomes across various settings [11,18]. Similarly, motivational interviewing, a patient-centered communication approach, has demonstrated effectiveness in addressing ambivalence and improving adherence to treatment plans by fostering collaboration and empathy between healthcare providers and patients [19]. Integrating these evidence-based models into ONS education strategies could improve patient understanding and compliance. However, to the best knowledge of the authors, such an approach is not documented in Croatian hospitals. This paper is based on the importance that patient education has in using the prescribed dose of ONSs. 

Therefore, this study aims to gain insight into currently hospitalized patients and investigate whether there is a statistically significant difference in the distribution of data between compliance with the intake of the prescribed dose at home in relation to the following:

- The source of information on how to consume ONSs (nurse, medical doctor (MD), nurse and MD, and other sources: Internet, nobody, TV)
- The time of the first ONS consumption (more than two years ago, half a year ago, one year ago, for the first time, less than half a year ago)

This investigation uses an instrument for examining patient attitudes and behaviors developed in accordance with the best practice of survey research [20,21].

## 2. Materials and Methods

### 2.1. Design and Participants

The participants of this survey were 120 patients hospitalized in Varaždin General Hospital, the Special Hospital for Chronic Diseases in Novi Marof, and the hospital for lung diseases and tuberculosis in Klenovnik in the Republic of Croatia. The survey was conducted as part of the Project ‘Acceptability and Adjustment of Dietary Supplements in Hospitalized Patients’ at University North—Department of Food Technology. The study design was in accordance with the EQUATOR NETWORK guideline, specifically the Standards for Reporting Qualitative Research (SRQR). The survey was administered by independent nurses who were not involved in the patients’ care or education, thereby ensuring unbiased data collection. All patients participated voluntarily, and informed consent was obtained prior to their inclusion in the study. It is important to note that no patients with restricted cognitive functions or illiteracy were included in the study (there were no such individuals among the patients), ensuring that all participants could fully comprehend and respond to the survey questions independently.

### 2.2. Research Procedure

For the purpose of this survey research, data were collected through an anonymous questionnaire in the Croatian language among hospitalized patients with their voluntary consent. This survey was approved by the General Hospital Ethics Committee and was conducted in the period between April and June 2024. The questionnaires were delivered to the General Hospital by the hospital’s head nurse, while ward nurses distributed them to patients, instructed them on how to fill them out, and later collected them. The completed questionnaires were then returned to the hospital’s head nurse who forwarded them to the research leader. The questionnaire consists of 20 multiple-choice questions divided into several groups from which data are obtained and processed regarding the education and awareness of the importance of and compliance with the intake of the recommended ONS dose. The data were anonymized by nurses to ensure the protection of patients’ personal information and to uphold confidentiality throughout the research process, while also enabling objective data analysis free from bias or identification of individual participants.

### 2.3. Statistical Processing

The obtained data were processed using Statistica 14.1.0.8 (Cloud Software Group Inc.) with descriptive and inferential statistical analyses. The Shapiro–Wilk test assessed the normality of distribution for quantitative variables, while the Levene test evaluated the homogeneity of variance. Quantitative variables were presented as means ± standard deviations and qualitative variables as absolute (n) and relative (%) frequencies. The nonparametric Mann–Whitney test compared differences in data distribution between groups, while the χ^2^ test examined differences in response frequencies.

To address concerns about the non-validated questionnaire, we conducted preliminary validation steps. Internal consistency was assessed using Cronbach’s alpha, yielding a score of 0.82, which is considered adequate for research purposes. Additionally, a post hoc power analysis was performed. For a medium effect size (w = 0.3) in a chi-square test with df = 1, α = 0.05, and N = 120, the achieved power was 0.88, indicating adequate statistical power for detecting significant differences.

For all hypothesis tests, a 95% confidence interval was used, with statistical significance set at *p* < 0.05 (two-tailed).

## 3. Results

**Figure 1:**
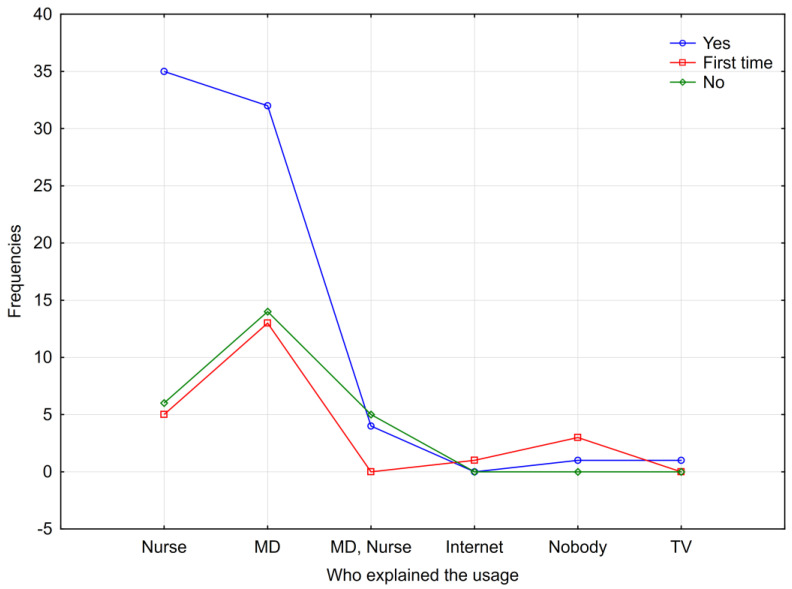
Data distribution between compliance with the intake of the recommended dose at home and sources of information on how to consume ONSs. Note—MD: medical doctor; TV: television.

There is a statistically significant difference in the distribution of data between compliance with the intake at home and the source of information on how to consume ONSs (*p* = 0.003—explanation via χ^2^ test) (Figure 1). 

**Figure 2:**
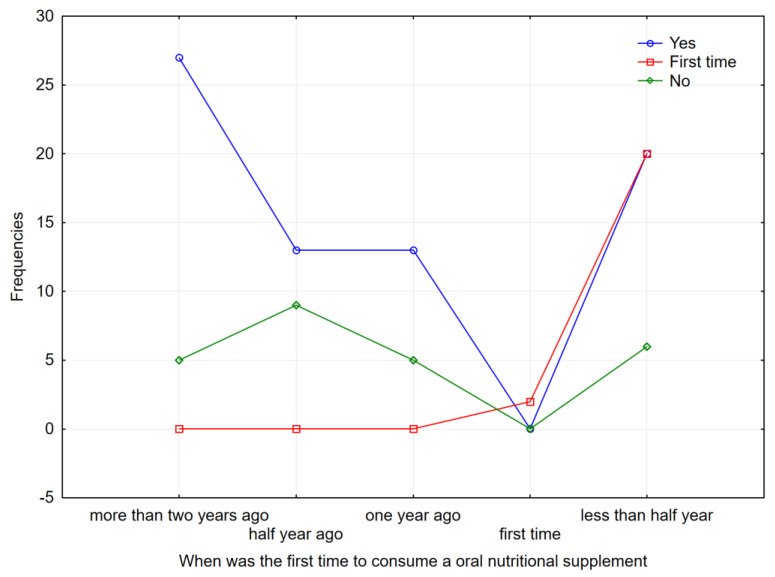
Data distribution between compliance with the recommended dose of ONSs at home and its first consumption. The time intervals at which the participants reported their usage of the recommended dosage of ONSs were set to more than two years, half a year ago, one year ago, first time, or less than half a year ago.

There is a statistically significant difference in the distribution of data between compliance with the intake at home and the first consumption of ONSs (*p* = 0.000—explanation via χ^2^ test) with a key time of 6 months (half a year) when most of them give up (Figure 2).

## 4. Discussion

In general, the use of recommended therapy is a fundamental aspect of healthcare and one of the most common and effective treatments for various health conditions [22]. However, it is not always easy for patients to comply with the prescribed regimen due to differences in patients’ characters and behaviors with regard to taking the prescribed therapy [23,24].

The results of this study are based on a separate study of 120 patients hospitalized in different General Hospital wards and their compliance with the consumption of prescribed doses of oral nutritional supplements (ONSs) in relation to the source of information and the initial time of ONS use. Dietary supplementation is sometimes very important for certain patients due to the patient’s diagnosis because the consumption of food rich in complex nutrients can lead to the disease being cured. The importance of compliance with the consumption of the recommended dose is supported by data that favor the reduction of time spent in hospital [25], hospitalization cost [26], percentage of complications [27], symptoms of depression [28], hospital readmission and reduction in mortality rates [29,30], and improvement in the percentage of muscle mass [31].

Motivational interviews and (patient/nurse) teaching are often used in the implementation of patient education for the assessment and evaluation components, while planning and implementation rely more on evidence-based strategies (evaluation of patient education materials). Nurses should provide simple, patient-centered, and interdisciplinary education to meet the needs of health literacy [11].

In the Republic of Croatia, ONSs are specifically categorized foods for special medical needs and are issued on a medical prescription with the recommendation of a specialist (who prescribes the daily dose and type of preparation, either during hospital stay or at home), and are often included in the basic or supplementary List of Medicines of the Croatian Health Insurance Fund (HZZO). They are sold exclusively in pharmacies. Food for specific medical needs is regulated by the Foods for Specific Groups Regulation [32]. The need for continued use is determined by a specialist from the contracted healthcare institution who is obliged to assess the effect and the need for continued use every 6 months.

Many patients refuse this type of dietary supplement [5]. One of the main reasons for this is the lack of patient education on the importance of ONS intake [33].

This study shows that there is a statistically significant difference in the distribution of data between compliance with the intake at home and the source of information on how to consume ONSs (*p* = 0.003—explained by χ^2^ test) (Figure 1).

Education on ONS consumption is crucial for positive patient outcomes. However, there are limitations regarding the optimal education of nurses and doctors on complying with therapy during hospitalization and with its continuation at home [34,35].

The conducted study, which included 120 patients (who complied, did not comply, or started with ONS intake for the first time in the hospital), shows that comprehensive education on ONS intake was carried out by nurses (38.33% or 46 patients), by doctors (49.17% or 59 patients), by nurses and doctors (7.50% or 9 patients), via the Internet (0.83% or 1 patient), by no one (3.33% or 4 patients), and via television (0.83% or 1 patient) (Table 1).

Some of the patients started consuming ONSs in the hospital for the first time (18.33%), where their source of information on the importance of ONS intake was a nurse (4.17%), a doctor (10.83%), a nurse and a doctor (0.00%), the Internet (0.83%), no one (2.50%), and television (0.00%). Compliance with the recommended dose advised by healthcare professionals ranges from 10.87% to 22.03%, while compliance that comes from other sources ranges from 75.00 to 100%. Looking at this through the percentage within each source, it is evident that 22.73% were educated by a nurse with respect to the recommended dose, as well as 59.09% who were educated by a doctor, 4.55% who were educated via the Internet, and 13.64% who were educated by no one. All data obtained range in value up to and around 50% of those educated by healthcare professionals (Table 1).

Some sources [36] provide insight into the fact that more than 90% of nurses believe it is important to provide information on the use of new medication, as well as on patients’ medical condition, on the use of medical resources, and on the evaluation of patients’ comprehension regarding ONS use.

Our study shows that a small percentage of patients educated by a nurse compared to other sources (22.73%), and only 10.87% educated by a nurse alone, who are now in hospital and have started consuming ONSs for the first time, complied with the recommended dose.

The cause of insufficient education is explained in reference [36] as the inability to devote more time to each patient individually. With 5–9 min spent per patient on the education front regarding how to use the medications, more than 80% of nurses consistently provide information about new medications and assess patient understanding using available resources.

Those who consume ONSs at home (60.83%) and respect the recommended dose were either educated by a nurse (29.17%), by a doctor (26.67%), by a nurse and a doctor (3.33%), via the Internet (0.00%), by no one (0.83%), or via television (0.83%). Compliance with the consumption of the recommended dose where the source of information was a healthcare professional ranges from 44.44% to 76.09%, while for other sources, the range is from 25.00 to 100%. Looking at this through the percentage within each source, it is evident that 47.95% of those educated by a nurse comply with the recommended dose, while 43.84% of compliance comes from those educated by a doctor, 5.48% from those educated by a nurse and a doctor, 1.37% from those educated by no one, and 1.37% from those educated by television. All of these percentages, in terms of information source, are below 50%, except for 76.09%, and refer to the nurse as the source of information.

Bowen et al. state that nurses have higher expectations in terms of how patients understand and follow instructions related to the use of medications [36]. However, some difficulties have been noticed in patient understanding of how medications should be used. Therefore, to improve it, nurses suggest the use of new sources of information on how medications ought to be used, which are tailored to patients.

The Joint Commission’s focus on improving transitional care describes models that include multidisciplinary methods for educating patients and healthcare professionals [37].

Some examples of strategies used by healthcare organizations for the improvement of patient education include the use of motivational interviews, the “teaching” method, and reminders [10]. These strategies are also used by healthcare professionals when prescribing medications to patients at hospital discharge.

Those who consume ONSs at home (20.83%) and do not comply with the recommended dose were educated by a nurse (5.00%), by a doctor (11.67%), by a nurse and a doctor (4.17%), via the Internet (0.00%), by no one (0.00%), and via television (0.00%). Non-compliance with the recommended dose advised by healthcare professionals as information sources ranges from 13.04% to 55.56%. Looking at this through the percentage within each source, it is evident that 24.00% of those educated by a nurse do not comply with the recommended doses, 56.00% of those educated by a doctor also do not, as well as 20.00% of those educated by both a nurse and a doctor. All of these are percentages that range in values less than 55%. The disparity in compliance between patients educated by doctors versus nurses may stem from differences in communication styles and time constraints. Doctors often have limited time for patient education due to heavy workloads, potentially leading to less personalized or comprehensive explanations. Nurses, on the other hand, typically spend more time with patients and may be better positioned to provide tailored education and address individual concerns. Additionally, patients might feel more comfortable asking questions or seeking clarification from nurses, leading to better understanding and compliance [18]. Further research is needed to explore the specific factors contributing to this disparity and develop strategies to improve doctor-led patient education.

In their work, the group of authors observes that there are targeted methods regarding education on the use of medications, which promote compliance. It is expected that patients will understand and accept healthcare education when healthcare professionals are focused on the patient and encourage them to share their views and treatment preferences. Studies have shown that shared decision-making improves compliance and, consequently, also improves disease control [18].

As a possibility for improvement, the provision of written materials should be included in the education of patients and their families, together with one-hour educational workshops [38]. However, there is no standardized approach to education on the use of medication, given that there is disagreement among service providers [39,40]. The World Health Organization for Patient Safety (2021–2030) is involved in this issue with the goal of reducing avoidable harm related to medications by at least 50% by 2030. It calls on all sectors of healthcare systems on a global scale to carefully review and improve their structures, processes, and methods [41].

There is also a statistically significant difference in the distribution of data between compliance with the intake at home and first consumption of ONSs (*p* = 0.000—explained by χ^2^ test) with the key time of 6 months (half a year) when most of them stop with ONS intake (Figure 2).

One of the reasons for this may be that the need for continued use is determined by a specialist from a contracted healthcare institution who is obliged to assess the effect and the need for continued use every 6 months. This suggests that patients did not continue with use due to their diagnosis, or that they stopped consuming ONSs due to insufficient education on the importance of ONS use. Only 58% of nurses believe that it is important to provide information about medications that are being prescribed again. However, one-third, or even less, specifically discussed and/or provided information about extended patient therapy [18]. 

Non-compliance with oral nutritional supplements (ONSs) is influenced by multiple underlying factors, including patient health literacy, socioeconomic barriers, and cultural perceptions. Low health literacy has been shown to reduce adherence rates, as patients [11] may struggle to understand the importance of ONSs and proper usage. Socioeconomic barriers, such as financial constraints or limited access to healthcare, can also hinder compliance [5]. Cultural beliefs may shape attitudes toward ONSs, with stigma or dietary restrictions affecting adherence [9]. Trust in healthcare providers plays a critical role, as [42] patients who trust their physicians are more likely to follow prescribed regimens. Additionally, the accessibility and format of educational materials significantly impact patient understanding and compliance; tailored, easy-to-understand resources in multiple formats can improve outcomes [1]. Addressing these factors through targeted interventions, such as nurse-led education programs or culturally sensitive materials, could enhance compliance and improve patient outcomes.

This qualitative study has some considerations to note. First, the data collection method provides a snapshot of participants’ experiences and perceptions at a single point in time, which may not capture the evolving nature of ONS compliance and education. Second, the study was conducted in a specific cultural and healthcare context within the Republic of Croatia, which may influence the transferability of findings to other settings. Finally, the self-reported data on ONS compliance and educational experiences may be subject to recall bias and social desirability effects, potentially influencing the depth and authenticity of responses.

Our findings highlight the need for standardized patient education on ONS use, emphasizing the role of nurses in this process. Future research should focus on developing and evaluating targeted educational interventions for both patients and healthcare professionals. The integration of nutrition-expert nurses, such as Clinical Nurse Specialists in Nutrition, could significantly enhance educational support and improve ONS compliance. Further studies should explore the impact of multidisciplinary approaches, including the involvement of dietitians and pharmacists, in optimizing ONS education and adherence.

## 5. Conclusions

Looking at the source of information, the highest percentage of patients who complied with the recommended dose of ONS intake at home (47.95%) received the information from a nurse.

The highest percentage of patients who complied with the recommended dose of ONS intake, and who started consuming ONSs for the first time in hospital, received the information from a doctor (59.09%). It is visible that, despite being in hospital, 13.64% of them were not educated from any source.

Furthermore, the highest percentage of patients who did not comply with the recommended dose of ONS intake at home (56.00%) received the information from a doctor, too.

Based on these findings, healthcare providers should implement a comprehensive university education that includes interactive content, videos, and personalized information tailored to patients’ specific ONS needs, complemented by nurse-led follow-up calls using structured protocols.

The obtained results indicate the necessity of changing the approach and form of patient education. New research through quantitative analysis (including nurses and patients) could lead to better quality patient education, as well as the development of a standardized framework and accessible tools for patient education.

The period of 6 months (half a year) is the time when most patients stop consuming ONSs. Further research should look into whether the reason for stopping consumption is due to the doctor’s recommendation or insufficient education on the importance of its use.

## Figures and Tables

**Table 1:** Display of data between compliance (YES) and non-compliance (NO) with the intake at home, as well as with the consumption for the first time in hospital, and sources of information on how to consume ONSs.

	S O U R C E						
Do You Comply with the Recommended Intake Dose?	Nurse	MD	MD; Nurse	Internet	Nobody	TV	Row Totals
**YES,** at home	35	32	4	0	1	1	73
Column Percent	76.09%	54.24%	44.44%	0.00%	25.00%	100.00%	
Row Percent	47.95%	43.84%	5.48%	0.00%	1.37%	1.37%	∑ = 100%
Total Percent	29.17%	26.67%	3.33%	0.00%	0.83%	0.83%	60.83%
**Now, for the first time** in hospital	5	13	0	1	3	0	22
Column Percent	10.87%	22.03%	0.00%	100.00%	75.00%	0.00%	
Row Percent	22.73%	59.09%	0.00%	4.55%	13.64%	0.00%	∑ = 100%
Total Percent	4.17%	10.83%	0.00%	0.83%	2.50%	0.00%	18.33%
**NO,** at home	6	14	5	0	0	0	25
Column Percent	13.04%	23.73%	55.56%	0.00%	0.00%	0.00%	
Row Percent	24.00%	56.00%	20.00%	0.00%	0.00%	0.00%	∑ = 100%
Total Percent	5.00%	11.67%	4.17%	0.00%	0.00%	0.00%	20.83%
**All Groups (∑ = 100%)**	46	59	9	1	4	1	120
**Total Percentage**	38.33%	49.17%	7.50%	0.83%	3.33%	0.83%	

**Note**—MD: medical doctor; TV: television.

## Data Availability

Patients/subjects were informed about the study and had the opportunity to make an independent decision about participating in it. The study was conducted anonymously.
